# Neuroendocrine marker staining pattern categorization of small‐sized pulmonary large cell neuroendocrine carcinoma

**DOI:** 10.1111/1759-7714.13202

**Published:** 2019-10-03

**Authors:** Kazuhiro Minami, Yugo Tanaka, Hiroyuki Ogawa, Naoe Jimbo, Wataru Nishio, Masahiro Yoshimura, Tomoo Itoh, Yoshimasa Maniwa

**Affiliations:** ^1^ Division of Thoracic Surgery Kobe University Graduate School of Medicine Kobe City Japan; ^2^ Department of Thoracic Surgery Hyogo Cancer Center Akashi City Japan; ^3^ Department of Diagnostic Pathology Kobe University Graduate School of Medicine Kobe City Japan

**Keywords:** Immunostaining, large cell neuroendocrine carcinoma, neuroendocrine markers, small cell lung carcinoma, small‐sized tumors

## Abstract

**Background:**

The aim of this study was to identify subgroups with good or bad prognosis in patients with pulmonary large cell neuroendocrine carcinoma (LCNEC) based on immunostaining patterns with neuroendocrine markers and compare them with small cell lung carcinoma (SCLC).

**Methods:**

From January 2001 to December 2017, of all patients with resected LCNEC and SCLC, we selected patients whose pathological tumor sizes were ≤30 mm in diameter (defined as small‐sized tumors) and who underwent complete resection with lymphadenectomy. We classified patients with small‐sized LCNEC (sLCNEC) into two subgroups based on immunostaining patterns with three neuroendocrine markers (chromogranin A, synaptophysin, and NCAM) and compared them to small‐sized SCLC (sSCLC).

**Results:**

A total of 48 patients with sLCNEC and 39 patients with sSCLC were enrolled. Of 48 patients with sLCNEC, 21 were categorized as the small‐sized triple‐positive group (sTP), whose patients were positive for the three neuroendocrine markers, and 27 patients were categorized as the small‐sized nontriple‐positive group (sNTP), whose patients were not positive for all three neuroendocrine markers. The percentage of lymph node metastasis was significantly lower in sNTP than in sTP and sSCLC. There was no significant difference in overall survival, but recurrence‐free survival (RFS) and tumor‐specific survival (TSS) were significantly poorer in sTP and sSCLC than in sNTP. Multivariate analysis revealed sTP and sSCLC were independent prognostic factors for poorer RFS and TSS than those of sNTP.

**Conclusions:**

The sNTP subgroup had a good prognosis and the sTP subgroup a poor prognosis. There were some similarities in clinicopathological features between sTP and sSCLC.

## Key points

### Significant findings of this study

Small‐sized LCNEC positive for three neuroendocrine markers (chromogranin A, synaptophysin, and NCAM) were associated with a poor prognosis and high rate of lymph node metastasis. Small‐sized LCNEC positive for three neuroendocrine markers had clinicopathological features similar to those of small‐sized SCLC.

### What this study adds

Small‐sized LCNEC could be classified into two subgroups based on immunostaining patterns with three neuroendocrine markers. In clinical practice, our study findings may provide new insights into treatment strategies for LCNEC, such as surgical indication and adjuvant chemotherapy.

## Introduction

Pulmonary neuroendocrine tumors represent approximately 20% of all lung cancers and can be subdivided into four major subtypes: typical carcinoid (TC), atypical carcinoid (AC), large cell neuroendocrine carcinoma (LCNEC), and small cell lung carcinoma (SCLC). Histologically, these tumors have neuroendocrine morphologies, such as organoid nesting, rosette‐like structures, and peripheral palisading patterns. TC and AC are categorized as low‐ and intermediate‐grade malignancy, whereas, LCNEC and SCLC are categorized as high‐grade malignancies.^1^, ^2^, ^3^

LCNEC, first proposed by Travis *et al*. in 1991,^4^ is a rare tumor known to be associated with shorter survival than that of other non‐small cell lung cancers (NSCLC),^5^, ^6^ whereas SCLC accounts for 13% of all lung carcinomas^7^ and is the most aggressive lung cancer. SCLC metastasizes lymph nodes and distant organs even in the early stage.^8^ The two types resemble each other both in clinical behavior, poor prognosis^9^, ^10^, ^11^ and genetic background.^12^, ^13^, ^14^, ^15^

Radical therapies for these tumors are considered to have limited applicability to small‐sized cases because of their rapid growth and early metastasis, but few studies have reported clinicopathological features in small‐sized LCNEC (sLCNEC) and small‐sized SCLC (sSCLC).

Regarding LCNEC, we previously reported a possible association between immunostaining patterns with three neuroendocrine markers (chromogranin A, synaptophysin, and neural‐cell adhesion molecule [NCAM]) and tumor response to chemotherapy. In that report, we categorized patients with LCNEC into two subgroups based on the immunostaining patterns of the three neuroendocrine markers and showed that perioperative chemotherapy might benefit the survival of patients with LCNEC if tumors were not immunoreactive to the three neuroendocrine markers.^16^ Our previous report also implied there might be some association between clinical outcomes in patients with LCNEC and the immunostaining patterns with the three neuroendocrine markers.

In this study, we tried to identify subgroups based on the immunostaining patterns with the three neuroendocrine markers, which had good or bad prognosis in sLCNEC. Additionally, we compared these subgroups to sSCLC to look for any new associations between LCNEC and SCLC.

## Methods

### Patients

From January 2001 to December 2017, 4865 consecutive patients with lung cancer underwent surgical resection at Kobe University Hospital and Hyogo Cancer Center. Of these, 138 (2.8%) patients were diagnosed as having LCNEC and 104 (2.1%) patients were diagnosed as having SCLC. We excluded patients whose pathological tumor size was >30 mm and who did not undergo complete anatomical resection (R0) with lymphadenectomy. We defined tumors that were ≤30 mm in pathological size as “small‐sized tumors.” We excluded patients whose tumor sizes were >30 mm because radical therapies for LCNEC and SCLC were expected to be limited in the small‐sized cases because of their rapid growth and early metastasis. We excluded patients who did not undergo lymphadenectomy because one of the study purposes was to clarify the frequency of lymph node metastasis in our patients. We finally selected 48 (1.0%) patients with sLCNEC and 39 (0.8%) patients with sSCLC. Surgical procedures were mainly lobectomies, but segmentectomies were performed in patients with impaired pulmonary function.

This study was approved by the Ethics Committee of Kobe University Hospital and Hyogo Cancer Center. Informed consent was obtained from all patients.

### Histopathology

The histological diagnoses of LCNEC were based on the criteria of the World Health Organization (2015)^2^: (i) neuroendocrine morphology, such as organoid nesting, trabecular growth, rosette‐like structures, and peripheral palisading pattern; (ii) moderate to abundant cytoplasm, low nuclear/cytoplasmic ratio and frequent nucleoli; (iii) high mitotic counts (>10 mitoses per 2 mm^2^); (iv) necrosis (usually large zone); and (v) neuroendocrine differentiation confirmed by using immunohistochemical markers, such as chromogranin A, synaptophysin, and NCAM. Neuroendocrine differentiation was confirmed by positive immunostaining for ≥1 of the three neuroendocrine markers mentioned above. Immunohistochemical stains were performed by using an anti‐synaptophysin antibody (monoclonal, MRQ‐40; Roche, Basel, Switzerland at Kobe University Hospital and monoclonal, 27G12; Nichirei, Tokyo, Japan at Hyogo Cancer Center), an anti‐chromogranin A antibody (monoclonal, DAK‐A3; Dako, Glostrup, Demark at Kobe University Hospital and Hyogo Cancer Center), and an anti‐NCAM antibody (monoclonal, MRQ‐42; Roche, at Kobe University Hospital and monoclonal, 1B6; Leica, New Castle, UK at Hyogo Cancer Center). The immunohistochemical staining of these markers was considered to be positive if >10% of the tumor cells were stained.

The histological diagnoses of SCLC were also based on the criteria of the World Health Organization (2015)^2^ relying on hematoxylin and eosin (H&E) staining.

In the present study, we included pure LCNEC and LCNEC combined with other NSCLC elements (combined LCNEC). Also, we included pure SCLC and SCLC combined with NSCLC elements (combined SCLC).

### Classification of LCNEC

We classified patients with sLCNEC into two subgroups based on our previous study^16^: patients who were positive for all three neuroendocrine markers were categorized as the small‐sized triple‐positive group (sTP) and those who were not positive for all three neuroendocrine markers were categorized as the small‐sized non‐triple‐positive group (sNTP).

### Patient characteristics

We compared clinicopathological characteristics among the sNTP, sTP, and sSCLC groups. We investigated the frequency of lymph node metastasis among the three groups and confirmed which group exhibited more aggressive behavior with early metastasis. We compared overall survival (OS), recurrence‐free survival (RFS) and tumor‐specific survival (TSS) among the three groups. Also, as subgroup analyses, we compared OS, RFS, and TSS between pure cases and combined cases as follows: pure sNTP vs. combined sNTP, pure sTP vs. combined sTP, pure sSCLC vs. combined sSCLC. Additionally, we performed multivariate analysis to identify the prognostic factors associated with survival after surgery.

Clinical information, including age, sex, smoking history, pathological stage, surgical procedure, adjuvant chemotherapy, pathological findings, and outcomes were retrieved from medical records. Pathological stage was determined according to the eighth Edition of the TNM Classification for Lung Cancer.^17^ OS was defined as the time from the date of operation to death from any cause or last follow‐up visit. RFS was defined as the time from the date of operation to relapse of disease or death from any cause. TSS was defined as the time from the date of operation to tumor‐related death, and patients without tumor‐associated deaths were censored.

### Statistical analysis

JMP, version 13, software (SAS Institute, Cary, NC, USA) was used to perform all statistical analyses. The differences in patients' characteristics among the groups were evaluated by analysis of variance or the chi‐square test or Fisher's exact test. The OS, RFS, and TSS were evaluated by Kaplan‐Meier survival analysis, and the log‐rank test was used to evaluate differences in the distributions. The prognostic factors for predicting survival after surgery were assessed by performing a multivariate analysis using Cox's proportional hazards model. *P*‐values of <0.05 were considered to be indicative of statistical significance, and a tendency was stated for *P*‐values of <0.10.

## Results

### Clinicopathological findings among sTP, sNTP, and sSCLC

**Figure 1:**
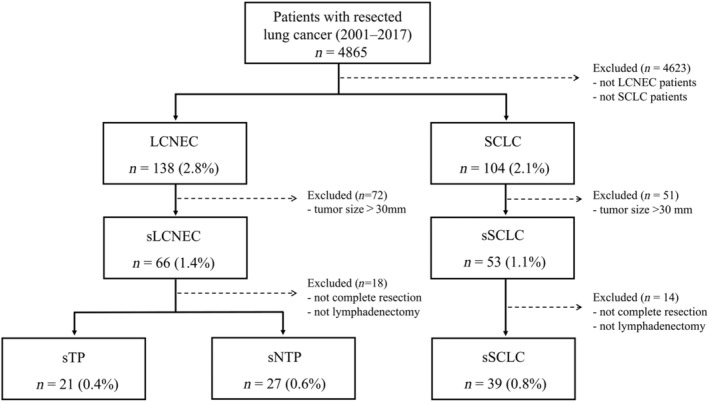
Flow diagram of patients with LCNEC and SCLC in this study. LCNEC, pulmonary large cell neuroendocrine carcinoma; SCLC, small cell lung carcinoma; sLCNEC, small‐sized LCNEC patients; sSCLC, small‐sized SCLC patients; sTP, small‐sized LCNEC patients who were positive for all three neuroendocrine markers (synaptophysin, chromogranin A, and NCAM); sNTP, small‐sized LCNEC patients who were positive for one or two of three neuroendocrine markers.

Among all patients with surgically resected lung cancer (*n* = 4865), 138 (2.8%) patients were diagnosed as having LCNEC, and 104 (2.1%) patients were diagnosed as having SCLC (Fig 1). Among the surgically resected LCNEC and SCLC patients, 66 (1.4%) were sLCNEC patients and 53 (1.1%) were sSCLC patients. Of the sLCNEC and sSCLC patients, those who did not undergo complete resection (R0) with hilar and mediastinal lymphadenectomy were excluded. Finally, 48 sLCNEC patients and 39 sSCLC patients were enrolled in this study.

**Figure 2:**
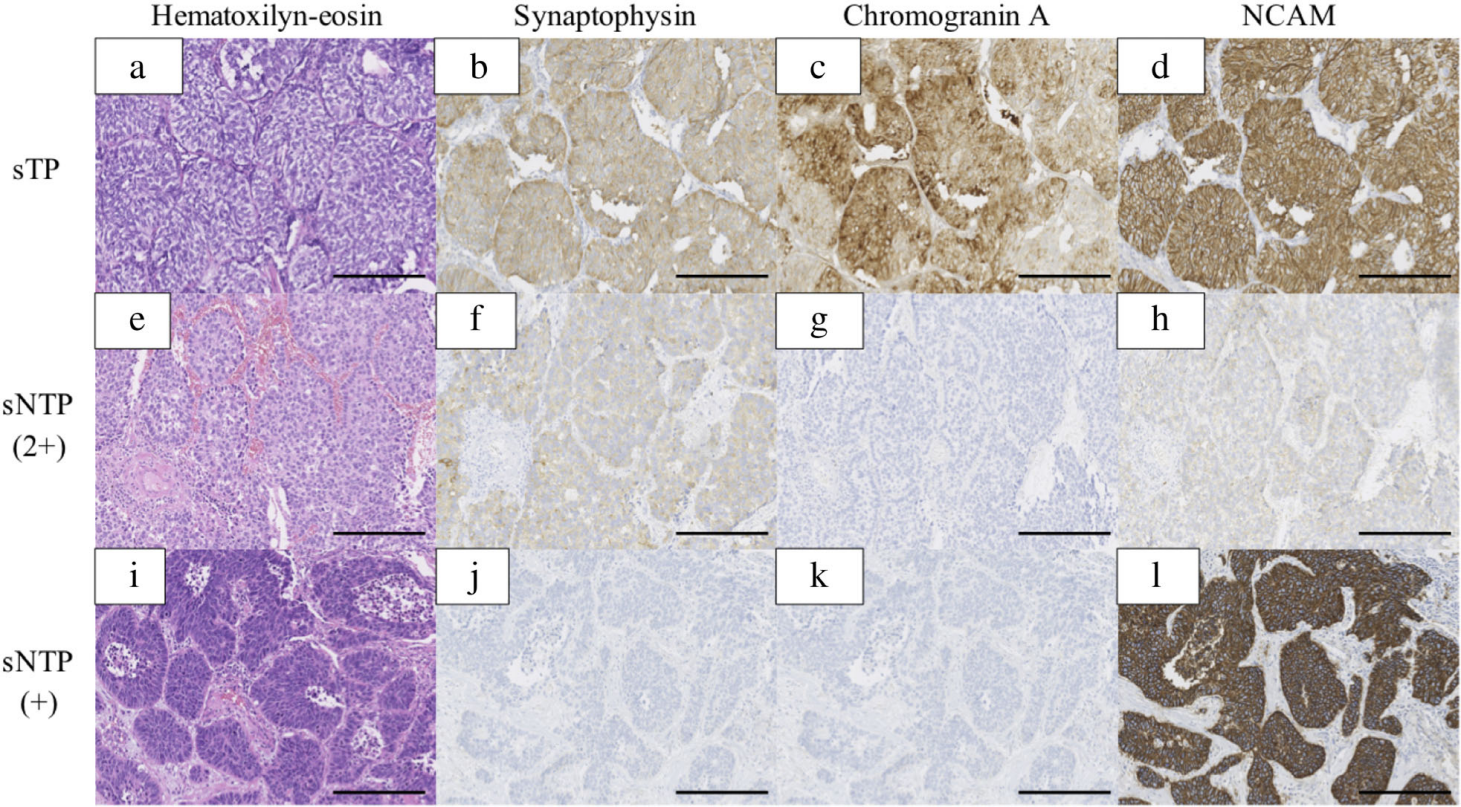
Representative pathological findings of sTP and sNTP. (**a**–**d**) sTP, (**e**–**h**) and (**i**–**l**) sNTP. (**a**, **e**, **i**) Hematoxylin‐eosin; (**b**, **f**, **j**) Synaptophysin; (**c**, **g**, **k**) Chromogranin A;(**d**, **h**, **l**) NCAM. Scale bar: 250 μm. sTP, small‐sized LCNEC patients who were positive for all three neuroendocrine markers (synaptophysin, chromogranin A and NCAM); sNTP, small‐sized LCNEC patients who were positive for one or two of the three neuroendocrine markers. +, positive for one of three neuroendocrine markers, 2+, positive for two of three neuroendocrine markers.

We classified the 48 sLCNEC patients into two subgroups according to staining patterns with the three neuroendocrine markers: sTP and sNTP. A total of 21 (0.4%) patients were categorized as sTP and 27 (0.6%) patients as sNTP. Figure 2 shows the representative pathological findings of sTP and sNTP. No obvious histological differences in H&E staining were found between them. Table 1 summarizes the clinicopathological characteristics among sNTP, sTP, and sSCLC. No significant differences were found in age, sex, tumor diameter, the presence of combined elements, the presence of necrosis, and surgical procedure among the three groups (*P* > 0.05), but significant differences were found in smoking history, pathological stages, lymphatic invasion (ly), mitotic counts, and adjuvant chemotherapy (*P* = 0.047, *P* = 0.018, *P* = 0.049, *P* = 0.024, and *P* = 0.0012, respectively).

**Table 1:** Clinicopathological characteristics among sNTP, sTP, and sSCLC

	sLCNEC				
Characteristics	All patients (*n* = 48)	sNTP (*n* = 27)	sTP (*n* = 21)	sSCLC (*n* = 39)	*P*‐value
Age (years)					0.31
Mean	67.7	68.6	66.5	69.1	
Range	30–81	51–81	30–78	56–84	
Sex					0.34
Male	40 (83)	24 (89)	16 (76)	29 (74)	
Female	8 (17)	3 (11)	5 (24)	10 (26)	
Smoking history					*
Current or former	44 (92)	27 (100)	17 (81)	37 (95)	
Never smoked	4 (8)	0 (0)	4 (19)	2 (5)	
Tumor diameter (mm)					0.65
Mean	22.1	22.2	21.9	23.4	
Range	8–30	8–30	12–30	15–30	
Combined elements					0.67
None (Pure)	37 (77)	21 (78)	16 (76)	33 (85)	
Combined	11 (23)	6 (22)	5 (24)	6 (15)	
Pathological stage					*
I	35 (73)	24 (89)	11 (52)	21 (54)	
II	6 (12)	2 (7)	4 (19)	11 (28)	
III	7 (15)	1 (4)	6 (29)	7 (18)	
Lymphatic invasion (ly)					*
Positive	34 (71)	16 (59)	18 (86)	32 (82)	
Negative	14 (29)	11 (41)	3 (14)	7 (18)	
Mitotic counts (/10 HPF)					*
Mean	58.2	50.8	67.8	65.4	
Range	12–119	16–100	12–119	20–150	
Necrosis					−
Present	48 (100)	27 (100)	21 (100)	39 (100)	
Absent	0 (0)	0 (0)	0 (0)	0 (0)	
Surgical procedure					0.53
Lobectomy	42 (87)	23 (85)	19 (90)	35 (90)	
Segmentectomy	6 (13)	4 (15)	2 (10)	4 (10)	
Adjuvant chemotherapy					**
Chemotherapy	11 (23)	4 (15)	7 (33)	23 (59)	
None	37 (77)	23 (85)	14 (67)	16 (41)	

* *P* < 0.05.

** *P* < 0.01.

Values are presented as *n* (%) or mean.

HPF, high‐powered fields; sLCNEC, small‐sized LCNEC patients; sNTP, small‐sized LCNEC patients who were positive for one or two of three the neuroendocrine markers; sSCLC, small‐sized SCLC patients; sTP, small‐sized LCNEC patients who were positive for all three neuroendocrine markers (synaptophysin, chromogranin A, and NCAM).

We evaluated differences in the frequency of lymph node metastasis among the three groups because there was a significant difference in pathological stages among them. The res3. The percentage of lymph node metastasis was significantly lower in sNTP than in sTP (11% and 48%, respectively, *P* < 0.01) and in sSCLC (11% and 44%, respectively, *P* < 0.01). The percentages of lymph node metastasis in sTP and sSCLC were similar, with no significant difference (48% and 44%, respectively, *P* = 0.76).

**Figure 3:**
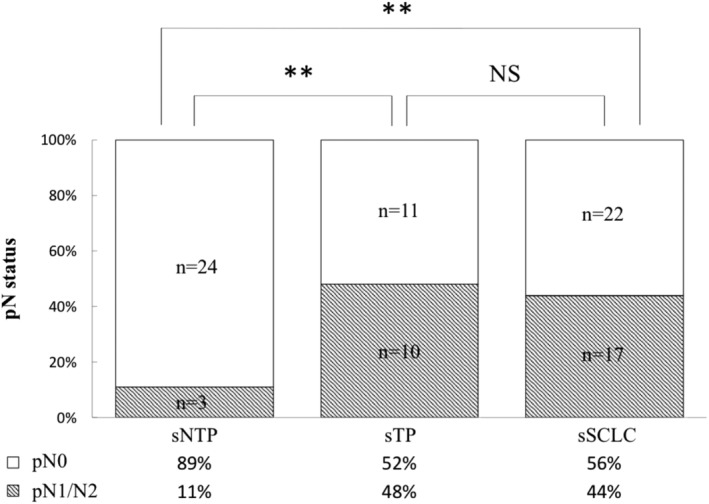
The rate of lymph node metastasis among sNTP, sTP, and sSCLC. NS, not significant; sNTP, small‐sized LCNEC patients who were positive for one or two of the three neuroendocrine markers; sSCLC, small‐sized SCLC patients; sTP, small‐sized LCNEC patients who were positive for all three neuroendocrine markers (synaptophysin, chromogranin A, and NCAM). **P < 0.01.

### Clinical outcomes among sTP, sNTP, and sSCLC

**Figure 4:**
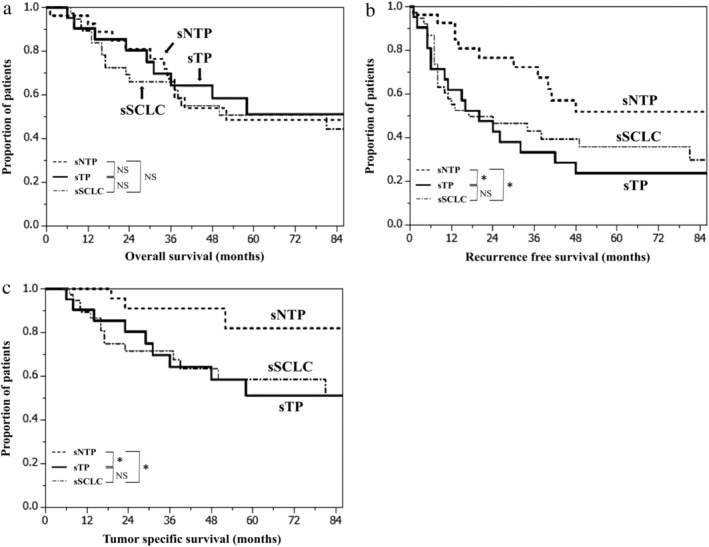
(**a**) Kaplan‐Meier curves of overall survival among sTP, sNTP, and sSCLC. (**b**) Kaplan‐Meier curves of recurrence‐free survival among sTP, sNTP, and sSCLC. (**c**) Kaplan‐Meier curves of tumor‐specific survival among sTP, sNTP, and sSCLC. NS, not significant; sNTP, small‐sized LCNEC patients who were positive for one or two of the three neuroendocrine markers; sSCLC, small‐sized SCLC patients; sTP, small‐sized LCNEC patients who were positive for all three neuroendocrine markers (synaptophysin, chromogranin A, and NCAM). *P < 0.05.

The median follow‐up time was 48 months (range, 6–138 months) in sTP, 40 months (range, 1–116 months) in sNTP, and 41 months (range, 7–130 months) in sSCLC. The five‐year OS rates, five‐year RFS rates, and five‐year TSS rates were 51.2%, 23.8%, and 51.2% in sTP, 48.6%, 51.9%, and 82.0% in sNTP and 50.8%, 35.9%, and 58.6% in sSCLC. There was no significant difference in OS among the three groups (Fig 4a), but RFS and TSS were significantly poorer in sTP than in sNTP (*P* = 0.026 and *P* = 0.038, respectively; Fig 4b,c). Additionally, RFS and TSS were significantly poorer in sSCLC than in sNTP (*P* = 0.036 and *P* = 0.026, respectively; Fig 4b,c). However, no significant differences in RFS and TSS were found between sTP and sSCLC (*P* = 0.654 and *P* = 0.943, respectively; Fig 4b,c). Also, no significant differences were observed between pure cases and combined cases (pure sNTP vs. combined sNTP, OS: *P* = 0.737, RFS: *P* = 0.996, TSS: *P* = 0.159, pure sTP vs. combined sTP, OS: *P* = 0.486, RFS: *P* = 0.966, TSS: *P* = 0.359, pure sSCLC vs. combined sSCLC, OS: *P* = 0.873, RFS: *P* = 0.839, TSS: *P* = 0.520).

### Multivariate analysis for OS, RFS, and TSS

To identify prognostic factors for OS, RFS, and TSS, multivariate analyses using six clinical factors (age, sex, surgical procedure, pN status, histology, and adjuvant chemotherapy; Tables 2, 3, 4) were performed. Among clinical factors, pN0 status was an independent favorable prognostic factor for OS, RFS, and TSS (OS: hazards ratio, 0.329, *P* = 0.0069; RFS: hazards ratio, 0.399, *P* = 0.0078; TSS: hazards ratio, 0.179, *P* = 0.0007). Prognosis was significantly poorer for RFS and TSS in sSCLC than in sNTP (RFS: hazards ratio 2.274, *P* = 0.036; TSS: hazards ratio, 5.349, *P* = 0.010). Although we observed tendencies toward inferior RFS and TSS in sTP compared with sNTP, the small number of subjects did not allow us to show statistically significant differences (RFS: hazards ratio, 2.069, *P* = 0.073; TS: hazards ratio, 3.460, *P* = 0.072). Prognosis for TSS was significantly better in the patients who underwent lobectomies than in those who underwent segmentectomies (hazards ratio, 0.154, *P* = 0.010).

**Table 2:** Multivariate analysis of prognostic factors influencing OS (Cox proportional hazards model)

Variable	HR	95% CI	*P*‐value
Age			
<75 vs. ≥75 years	0.688	0.339–1.433	0.311
Sex			
Male vs. female	1.483	0.643–3.882	0.369
Surgical procedure			
Lobectomy vs. segmentectomy	0.564	0.200–2.031	0.348
pN status			
pN0 vs. pN1/N2	0.329	0.149–0.734	**
Histology			
sSCLC vs. sNTP	1.189	0.537–2.667	0.669
sTP vs. sNTP	0.662	0.258–1.625	0.371
sSCLC vs. sTP	1.796	0.781–4.441	0.171
Adjuvant chemotherapy			
Surgery with chemotherapy vs. surgery alone	0.533	0.2229–1.174	0.120

** *P* < 0.01.

CI, confidence interval; HR, hazard ratio; OS, overall survival; sNTP, small‐sized LCNEC patients who were positive for one or two of the three neuroendocrine markers; sSCLC, small‐sized SCLC patients.; sTP, small‐sized LCNEC patients who were positive for all three neuroendocrine markers (synaptophysin, chromogranin A, and NCAM).

**Table 3:** Multivariate analysis of prognostic factors influencing RFS (Cox proportional hazards model)

Variable	HR	95% CI	*P*‐value
Age			
<75 vs. ≥75 years	0.851	0.448–1.670	0.632
Sex			
Male vs. female	1.313	0.654–2.880	0.458
Surgical procedure			
Lobectomy vs. segmentectomy	0.633	0.262–1.892	0.379
pN status			
pN0 vs. pN1/N2	0.399	0.205–0.782	**
Histology			
sSCLC vs. sNTP	2.274	1.055–5.101	*
sTP vs. sNTP	2.069	0.935–4.682	†
sSCLC vs. sTP	1.099	0.559–2.219	0.786
Adjuvant chemotherapy			
Surgery with chemotherapy vs. surgery alone	0.664	0.329–1.324	0.245

* *P* < 0.05.

** *P* < 0.01.

† *P* < 0.1.

CI, confidence interval; HR, hazard ratio; RFS, recurrence‐free survival; sNTP, small‐sized LCNEC patients who were positive for 1 or 2 of the three neuroendocrine markers; sSCLC, small‐sized SCLC patients.; sTP, small‐sized LCNEC patients who were positive for all three neuroendocrine markers (synaptophysin, chromogranin A, and NCAM).

**Table 4:** Multivariate analysis of prognostic factors influencing TSS (Cox proportional hazards model)

Variable	HR	95% CI	*P*‐value
Age			
<75 vs. ≥ 75 years	0.811	0.324–2.135	0.663
Sex			
Male vs. female	1.248	0.484–3.739	0.661
Surgical procedure			
Lobectomy vs. segmentectomy	0.154	0.046–0.609	*
pN status			
pN0 vs. pN1/N2	0.179	0.061–0.487	**
Histology			
sSCLC vs. sNTP	5.349	1.454–26.25	*
sTP vs. sNTP	3.460	0.901–17.30	†
sSCLC vs. sTP	1.546	0.588–4.303	0.380
Adjuvant chemotherapy			
Surgery with chemotherapy vs. surgery alone	0.100	0.146–1.174	0.100

* *P* < 0.05.

** *P* < 0.01.

† *P* < 0.1.

CI, confidence interval; HR, hazard ratio; sNTP, small‐sized LCNEC patients who were positive for 1 or 2 of the three neuroendocrine markers; sSCLC, small‐sized SCLC patients.; sTP, small‐sized LCNEC patients who were positive for all three neuroendocrine markers (synaptophysin, chromogranin A, and NCAM); TSS, tumor‐specific survival.

## Discussion

In this study, we found that the sNTP subgroup had a good prognosis and the sTP subgroup had a bad prognosis in sLCNEC. Moreover, sTP was similar to sSCLC in clinicopathological features, such as the frequency of lymphatic invasion and lymph node metastasis, mitotic counts, survival curves, and poor prognosis (Figs 3, 4, Tables 1, 3, 4).

Two studies have recently reported the classification of LCNEC into subgroups using next generation sequencing.^13^, ^15^

Rekhtman *et al*. classified LCNEC into SCLC‐like, NSCLC‐like, and carcinoid‐like subset based on gene mutational profiles using custom targeted sequencing panels.^13^ In their study, 40% of LCNEC showed SCLC‐like gene mutational profile, characterized by coalteration of *TP53* and *RB1* (retinoblastoma‐related gene 1), 56% of LCNEC exhibited NSCLC‐like gene mutational profile, characterized by the lack of *TP53* and *RB1* coalteration and the presence of *STK11*/*KRAS* mutations.

Following the report by Rekhtman *et al*. George *et al*. classified LCNEC into two subtypes, named type I LCNEC and type II LCNEC, not only based on gene mutational profiles (such as *TP53* or *RB1*) but also on neuroendocrine gene expression profiles using the RNA sequencing expression data on 69 LCNECs and 110 SCLCs.^15^ These authors found that despite their gene mutational patterns, type I LCNEC lacking of *TP53* and *RB1* coalteration exhibited high expression of neuroendocrine genes with closest similarity to those of SCLC, and type II LCNEC with coalteration of *TP53* and *RB1* revealed reduced expression of neuroendocrine genes.

Our classification of LCNEC was based on neuroendocrine profiles. Our result was consistent with that of the study by George *et al*. 15 where LCNEC could be classified into two subgroups based on immunostaining patterns with the three neuroendocrine markers and sTP, which exhibited high expression of neuroendocrine markers, which were similar to sSCLC in clinicopathological features. Further studies are needed to investigate the relationship between gene mutational profiles and neuroendocrine gene expression profiles.

Although the two studies did not show significant differences in clinicopathological features among LCNEC subtypes and SCLC, we showed that pathological stages, frequency of lymphatic invasion, mitotic counts, frequency of lymph node metastasis, RFS, and TSS were significantly different among sNTP, sTP, and sSCLC. The reason why significant differences in clinicopathological features were observed in our study might be that our study focused on small‐sized tumors; LCNEC and SCLC are categorized as high‐grade malignancies, and in studies including large tumors it is expected that it will be difficult to show clinicopathological differences.

Our results implied that high expression of neuroendocrine markers resulted in high malignancy and poor outcome; however, the reason why neuroendocrine marker expression affected malignancy and prognosis of LCNEC remains unclear. Two other studies have reported associations between neuroendocrine markers and clinical outcomes in patients with high‐grade neuroendocrine carcinoma (HGNEC). Eichhorn *et al*. reported that simultaneous expression of both NCAM and chromogranin A was associated with poor outcome and high risk of recurrence in LCNEC patients.^18^ Hamanaka *et al*. reported that a subset of SCLC with low neuroendocrine expression showed better prognosis than a subset with high neuroendocrine expression.^19^ However, neither of them explained why neuroendocrine expression was correlated with malignancy. Further studies are needed to answer this question.

Only one study reported clinicopathological differences between sLCNEC and sSCLC. Isaka *et al*. compared 10 patients with sLCNEC to 18 patients with sSCLC.^20^ They reported that sSCLC showed poorer prognosis with early lymph node involvement and frequent postoperative recurrence than that of sLCNEC. Our study differed from their study by the smaller number of patients and because they did not classify sLCNEC into subgroups. In our study, we found that sLCNEC could be classified into subgroups with good or bad prognosis, and the clinicopathological features of the latter (sTP) were similar to those of sSCLC.

In our previous study, we compared NTP and TP (not limited to small‐sized tumors), and found that perioperative chemotherapy may benefit the survival of patients with NTP more than the survival of patients with TP^16^; however, our previous study did not show other clinocopathological differences between them, although our current study, which was limited to small‐sized tumors showed clinocopathological differences between sNTP and sTP such as pathological stages, the frequency of lymphatic invasion, mitotic counts, the frequency of lymph node metastasis and prognosis. This could mean that by limiting the study to small‐sized tumors, we confirmed the biological difference between sTP and sNTP.

In clinical practice, our study findings might provide new insights into treatment strategies for LCNEC, such as surgical indication and adjuvant chemotherapy. For example, if a patient is diagnosed as having sTP and hilar lymph node metastasis is suspected at the preoperative examination, we might exclude patients from surgical indication according to the SCLC‐based treatment strategy,^21^, ^22^, ^23^ although the surgical indication for patients with LCNEC is not often limited in stage I cases^24^, ^25^ (Fig S1). An adjuvant chemotherapy regimen for patients with LCNEC might be determined on the basis of immunostaining patterns with the three neuroendocrine markers depending on the subtype.

There were several limitations in this study. First, this was a retrospective study in a small number of subjects as it is difficult to plan a large‐scale study considering that the surgical indications for patients with HGNEC are limited because of rapid tumor growth. Second, although prognosis was significantly better for RFS and TSS in patients with sNTP than in patients with sTP or sSCLC, there were no significant differences in OS among them. This finding might be explained by the following: the number of tumor‐associated deaths was significantly less in patients with sNTP than in patients with sNTP or sSCLC (Table S1). Moreover, adjuvant chemotherapy was less likely to be administered in patients with sNTP in the present study (Table 1). Third, the anti‐synaptophysin antibody (clone; MRQ‐40 or 27G12) and anti‐NCAM antibody (clone; MRQ‐42 or 1B6) used in this study were different at each institution, but these clones are commonly used in pathological diagnosis worldwide, and the percentages of positive immunohistochemical staining for the three neuroendocrine markers were not very different between the two institutions (data not shown). This suggests that we could achieve the same results in different institutions.

In conclusion, we classified patients with sLCNEC into two subgroups according to three neuroendocrine markers which are necessary for diagnosis of LCNEC. Patients with sNTP showed significantly lower frequency of lymph node metastasis and significantly better RFS and TSS than those of patients with sTP or sSCLC. Patients with sTP had clinicopathological features similar to those of patients with sSCLC, such as the frequency of lymph node metastasis and poor outcome. Further studies, including genetic analysis and molecular considerations, are needed to obtain better understanding of the nature of high neuroendocrine tumors.

## Disclosure

The authors declare that they have no conflicts of interest related to this study.

## Supporting information


**Table S1** Cause of death and recurrence rate after surgery among sNTP, sTP, and sSCLC.

**Figure S1** Treatment strategy for LCNEC based on the immunostaining patterns. NSCLC, non‐small cell lung carcinoma; sLCNEC, small‐sized LCNEC patients; sNTP, small‐sized LCNEC patients who were positive for one or two of the three neuroendocrine markers; sSCLC, small‐sized SCLC patients; sTP, small‐sized LCNEC patients who were positive for all three neuroendocrine markers (synaptophysin, chromogranin A, and NCAM).
