# Supplementary material for: Neuroendocrine marker staining pattern categorization of small‐sized pulmonary large cell neuroendocrine carcinoma
Source: Thorac Cancer. 2019 Oct 3;10(11):2152–60. doi: 10.1111/1759-7714.13202 (PMC6825905; doi:10.1111/1759-7714.13202)
Supplement: Supplementary file 1 — Table S1 Cause of death and recurrence rate after surgery among sNTP, sTP, and sSCLC. [file TCA-10-2152-s001.docx]

**Supporting information**

| **Table S1** | | | | | | | | |  |
| --- | --- | --- | --- | --- | --- | --- | --- | --- | --- |
| Cause of death and recurrence rate after surgery among sNTP, sTP, and sSCLC | | | | | | | | | |
|  |  |  |  |  |  |  |  |  |  |
|  | sLCNEC | | | | | |  |  |  |
| Characteristics | All patients (n = 48) | | sNTP (n = 27) | | sTP (n = 21) | | sSCLC (n = 39) | | *P* value |
| Deaths | 23 | (48) | 14 | (52) | 10 | (48) | 19 | (49) | *** |
| Tumor-specific death | 12 | (25) | 3 | (11) | 10 | (48) | 14 | (36) |  |
| Other cause of death | 11 | (23) | 11 | (41) | 0 | (0) | 5 | (13) |  |
|  |  |  |  |  |  |  |  |  |  |
| Recurrence after surgery | 21 | (44) | 6 | (22) | 16 | (76) | 19 | (49) | *** |
| Values are presented as n (%)． sLCNEC: small-sized LCNEC patients; sSCLC: small-sized SCLC patients; sTP: small-sized LCNEC patients who were positive for all three neuroendocrine markers (synaptophysin, chromogranin A, and NCAM); sNTP: small-sized LCNEC patients who were positive for 1 or 2 of the three neuroendocrine markers. ***: *P* < 0.001. | | | | | | | | | |
|  |  |  |  |  |  |  |  |  |  |
