# Supplementary figures and images for: Neuroendocrine marker staining pattern categorization of small‐sized pulmonary large cell neuroendocrine carcinoma
Source: Thorac Cancer. 2019 Oct 3;10(11):2152–60. doi: 10.1111/1759-7714.13202 (PMC6825905; doi:10.1111/1759-7714.13202)

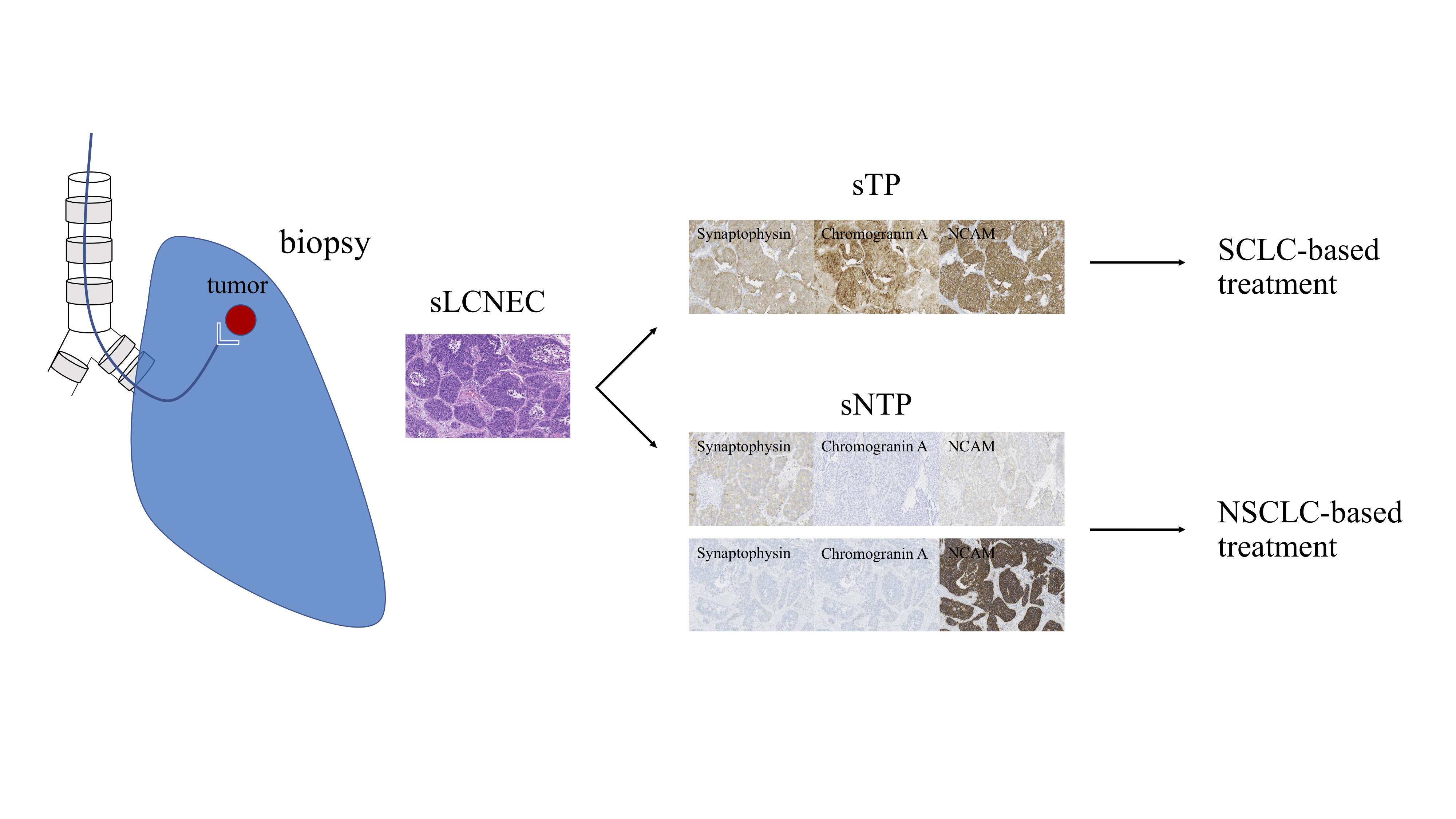

Supplement: Supplementary file 2 — Figure S1 Treatment strategy for LCNEC based on the immunostaining patterns. NSCLC, non‐small cell lung carcinoma; sLCNEC, small‐sized LCNEC patients; sNTP, small‐sized LCNEC patients who were positive for one or two of the three neuroendocrine markers; sSCLC, small‐sized SCLC patients; sTP, small‐sized LCNEC patients who were positive for all three neuroendocrine markers (synaptophysin, chromogranin A, and NCAM). [file TCA-10-2152-s002.jpg]
